# Detection of mandibular fractures on panoramic radiographs using deep learning

**DOI:** 10.1038/s41598-022-23445-w

**Published:** 2022-11-15

**Authors:** Shankeeth Vinayahalingam, Niels van Nistelrooij, Bram van Ginneken, Keno Bressem, Daniel Tröltzsch, Max Heiland, Tabea Flügge, Robert Gaudin

**Affiliations:** 1 Department of Oral and Maxillofacial Surgery, Radboud University Nijmegen Medical Centre, Postal Number 590, P.O. Box 9101, 6500 HB Nijmegen, The Netherlands; 2 Department of Oral and Maxillofacial Surgery, Charité – Universitätsmedizin Berlin, Corporate Member of Freie Universität Berlin and HumboldtUniversität zu Berlin, Augustenburger Platz 1, 13353 Berlin, Germany; 3 Einstein Center for Digital Future, Wilhelmstraße 67, Berlin, Germany; 4 Berlin Institute of Health at Charité – Universitätsmedizin Berlin, Charitéplatz 1, 10117 Berlin, Germany

**Keywords:** Health care, Dentistry, Diagnosis, Fracture repair, Computational science

## Abstract

Mandibular fractures are among the most frequent facial traumas in oral and maxillofacial surgery, accounting for 57% of cases. An accurate diagnosis and appropriate treatment plan are vital in achieving optimal re-establishment of occlusion, function and facial aesthetics. This study aims to detect mandibular fractures on panoramic radiographs (PR) automatically. 1624 PR with fractures were manually annotated and labelled as a reference. A deep learning approach based on Faster R-CNN and Swin-Transformer was trained and validated on 1640 PR with and without fractures. Subsequently, the trained algorithm was applied to a test set consisting of 149 PR with and 171 PR without fractures. The detection accuracy and the area-under-the-curve (AUC) were calculated. The proposed method achieved an F1 score of 0.947 and an AUC of 0.977. Deep learning-based assistance of clinicians may reduce the misdiagnosis and hence the severe complications.

## Introduction

Mandibular fractures are among the most frequent facial traumas in oral and maxillofacial surgery, accounting for 56.9%^1^. Vehicle accidents, body assaults, sports injuries, and falls are the leading causes of mandibular fractures^2,3^. Depending on the severity and complexity of these fractures, treatment ranges from nonoperative management to closed reposition with maxillomandibular fixation to open reduction with internal fixation^4^. An accurate diagnosis and appropriate treatment plan are vital for achieving optimal re-establishment of occlusion, function, and facial aesthetics^5–7^.

Panoramic radiographs (PR) are often the first-level imaging technique in facial trauma patients^8^. Even though PRs aid clinicians in diagnosis, decision-making and planning, there are some limitations to this imaging technique. The lack of three-dimensionality, homogeneity and potential artifacts in regions of interest impede clinicians’ accurate detection of fractures^9,10^. The misdetection of mandibular fractures can lead to severe complications such as malocclusion, non-union, osteomyelitis, haemorrhage, and airway obstruction^7,11,12^. An automated assistance system may reduce the number of complications, allowing a more reliable and accurate assessment of facial fractures, especially in the hands of less experienced professionals.

With advancements in artificial intelligence, deep learning algorithms have been adopted in computer-aided detection and diagnosis (CAD)^13^. Deep learning algorithms such as convolutional neural networks (CNNs) and vision transformers transform input data (e.g., images) to outputs (e.g., disease present/absent) while learning the higher-level features progressively^13,14^. Vision transformers were recently introduced in computer vision^15^. These architectures are based on a self-attention mechanism that learns the relationships between the elements of a sequence and can be efficiently scaled to high-complexity models and large-scale datasets. Different authors have proposed their implementation of a transformer model applied to vision, obtaining promising results in object detection^16^, segmentation^17^, video analysis^18^, and image generation^19,20^.

In oral and maxillofacial surgery, few studies have explored the capability of CNNs to detect mandibular fractures on PR automatically^10,21^. However, none of the studies has explored the detection and multi-classification (e.g., condyle, coronoid, ramus, paramedian, median, angle) of mandibular fractures on PR. This study aims to develop and evaluate vision transformers for mandibular fracture detection and classification as a fundamental basis for improved and more time-efficient diagnostics in emergency departments.

## Material and methods

### Data

In the present study, 6404 PR of 5621 patients (1624 PR with fractures, 4780 PR without fractures) were randomly collected from the Department of Oral and Maxillofacial Surgery of Charité Berlin, Germany. Median age of patients was 42 years, interquartile range (IQR) 34 years, age range of 18–99 years. 3728 patients were male (median age 38 years, IQR 32, age range 18–92 years) and 2438 were female (median age 48 years, IQR 35, age range 18–99 years). The accumulated PR were acquired with two different devices (Orthophos XG, Elite CR) from two different manufacturers (Sirona, Kodak). Blurred and incomplete PR(s) were excluded from further analyses. This study has been conducted in accordance with the code of ethics of the World Medical Association (Declaration of Helsinki). Informed consent was obtained from all participants. The approval of this study was granted by the Institutional Review Board (Charité ethics committee EA2/089/22).

### Data annotation

The present mandibular fractures on PR were classified and annotated with a bounding box based on electronic medical records (EMR), including clinical and additional radiological examinations. The bounding box must fully contain the fracture line. In dislocated fractures, the bounding box should include both fracture margins (e.g., condyle and ramus) and the fracture gap. All annotated PRs were subsequently reviewed and revised by three clinicians (RG, DT, TF). Disagreements were resolved by consensus. The three reviewers had at least five years of clinical experience. Each clinician was instructed in the labelling task using a standardized protocol prior to the annotation and reviewing process^9,22^.

### Model architecture

**Figure 1:**
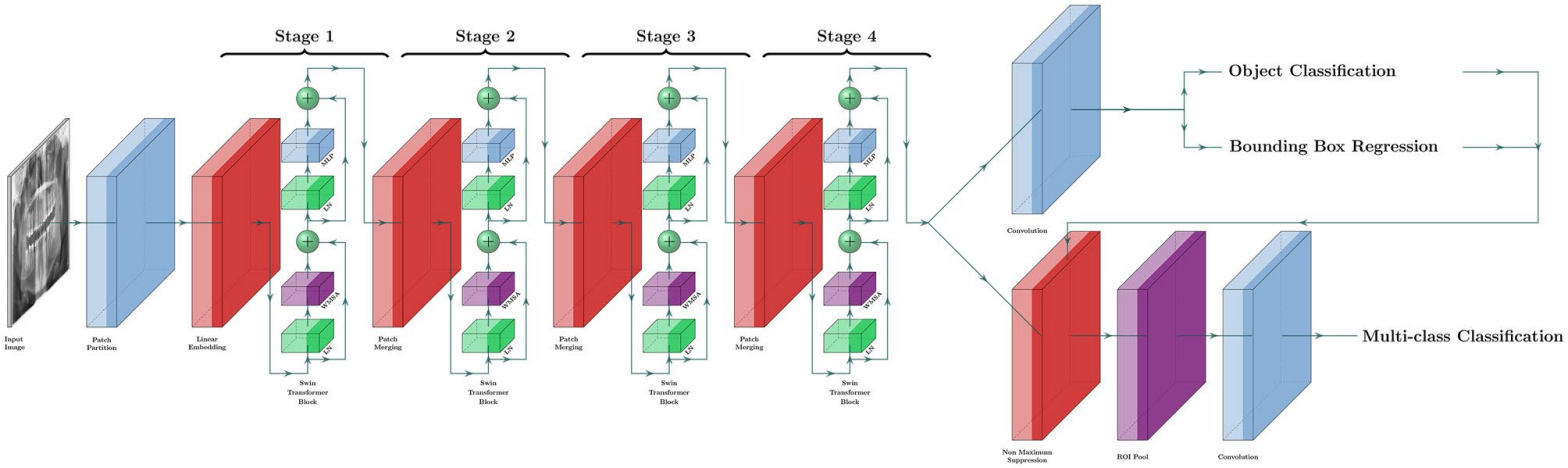
Faster R-CNN with Swin-Transformer.

A Faster R-CNN^23^ with Swin-Transformer^24^ was used in this study (Fig. 1). Faster R-CNN is a network for object detection that outputs bounding boxes around objects of interest with class labels (e.g., condyle, coronoid, ramus, paramedian, median, angle) and probabilities. Faster R-CNN contains a region proposal module that identifies the regions the classifier should consider^23^. In this module, the Swin-Transformer was used as a backbone. This is a recently proposed powerful vision transformer architecture characterized by shifting the window partitions between consecutive self-attention layers. The shifted windows connect with preceding layers’ windows, increasing the modelling power in an efficient manner^24^.

### Model training

The annotated PR with fractures were divided with multi-label stratification into three splits, 1310 for training, 165 for validation, and 149 for testing, based on the fracture’s subregion (condyle, coronoid, ramus, paramedian, median, angle). Specifically, the training set consisted of 364 angle fractures, 492 condyle fractures, 61 coronoid fractures, 187 median fractures, 487 paramedian fractures, and 180 ramal fractures. The validation set had 44 angle fractures, 61 condyle fractures, 8 coronoid fractures, 23 median fractures, 58 paramedian fractures and 22 ramal fractures. In contrast, the test set had 45 angle fractures, 65 condyle fractures, 7 coronoid fractures, 24 median fractures, 60 paramedian fractures and 21 ramal fractures. The control PR without fractures were randomly sampled and divided over the three splits. The validation split was used to select an optimal model performance during training and hyperparameter selection, while the held-out test split was used to evaluate the model performance after training and hyperparameter selection.

All PR image intensity values were normalized by subtracting the mean and dividing by its standard deviation. Random horizontal flipping and resizing were applied to augment the effective dataset.

The model optimization used the mini-batch stochastic gradient descent optimizer at an initial learning rate of 0.001, divided by ten after epochs 8 and 11. The Faster R-CNN architecture used a semi-supervised binary cross-entropy loss function to optimize the classification branch and the mean squared error loss function to optimize the detection branch. The model was trained over 12 epochs with a mini-batch size of 2, a momentum of 0.9, and a weight decay of 1e−4. The model was implemented in PyTorch and trained on a single NVIDIA^®^ Titan V GPU 12G^24^.

### Model inference

Twenty-two variants of each PR were created using horizontal flipping and resizing. The model predictions from all 22 variants were combined for the final inference result to increase the model’s sensitivity (test-time augmentation). The model’s confidence of fracture detection was computed as the maximum predicted intersection-over-union (IoU) of all bounding boxes, and a confidence threshold of 0.47 was applied. The confidence threshold was previously determined during training and validation. For the binary detection task, the fractures from all subregions were aggregated into a general fracture class.

### Statistical analysis

The model predictions on the test set were compared to the reference annotations using scikit-learn (version 1.1.1.). Object detection metrics are reported as follows for the test set: precision = $\frac{TP}{TP+FP}$, F1 score = $\frac{2TP}{2TP+FP+FN}$ (also known as the Dice-coefficient), recall = $\frac{TP}{TP+FN}$ (also known as sensitivity). TP, TN, FP, and FN denote true positives, true negatives, false positives, and false negatives, respectively. Furthermore, the area under the curve receiver operating characteristics curve (AUC), the precision-recall curve (AP), and the confusion matrix are presented^9,22^.

## Results

**Figure 2:**
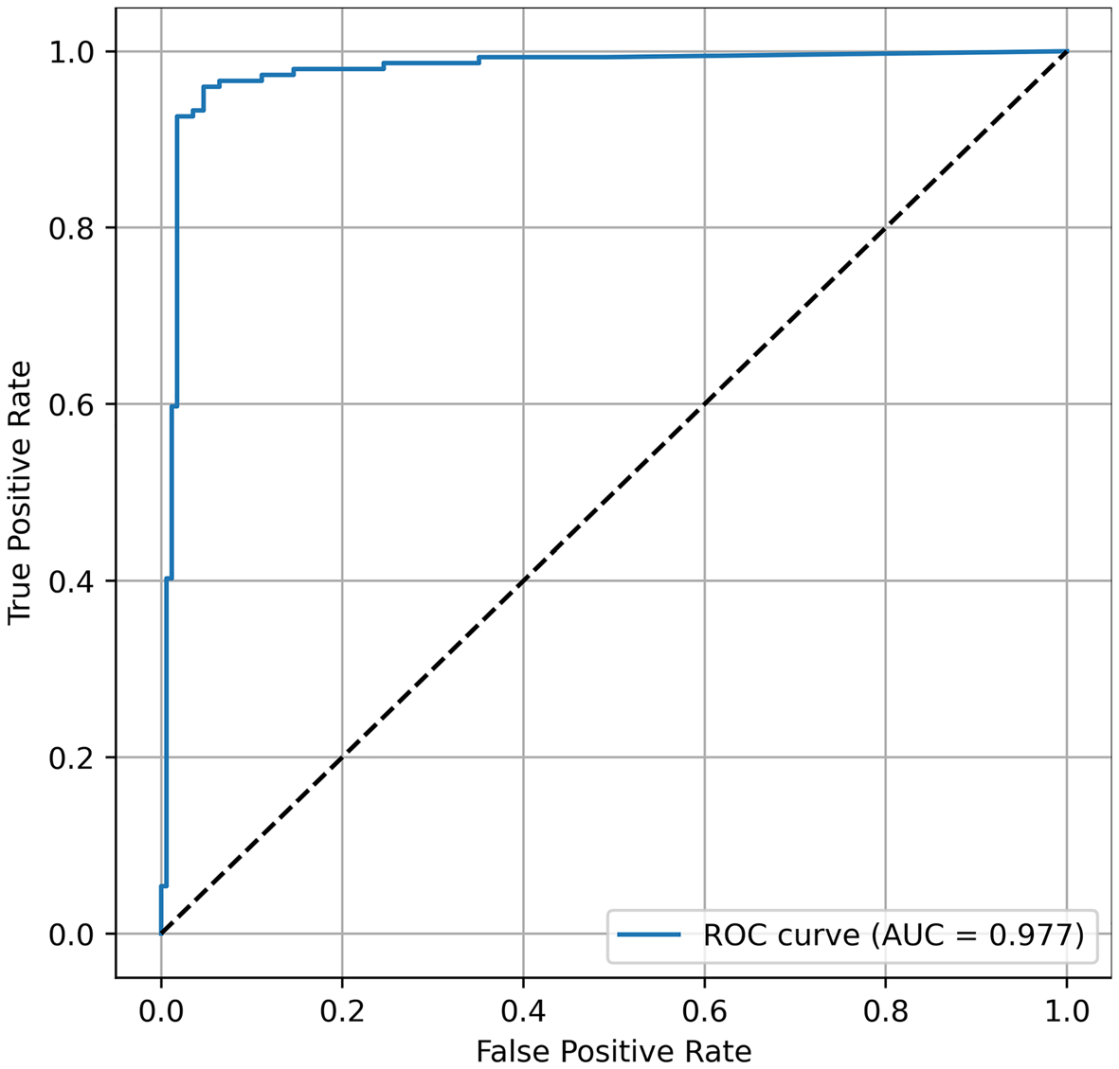
Area-under-the-curve-receiver-operating-characteristics-curve.

**Figure 3:**
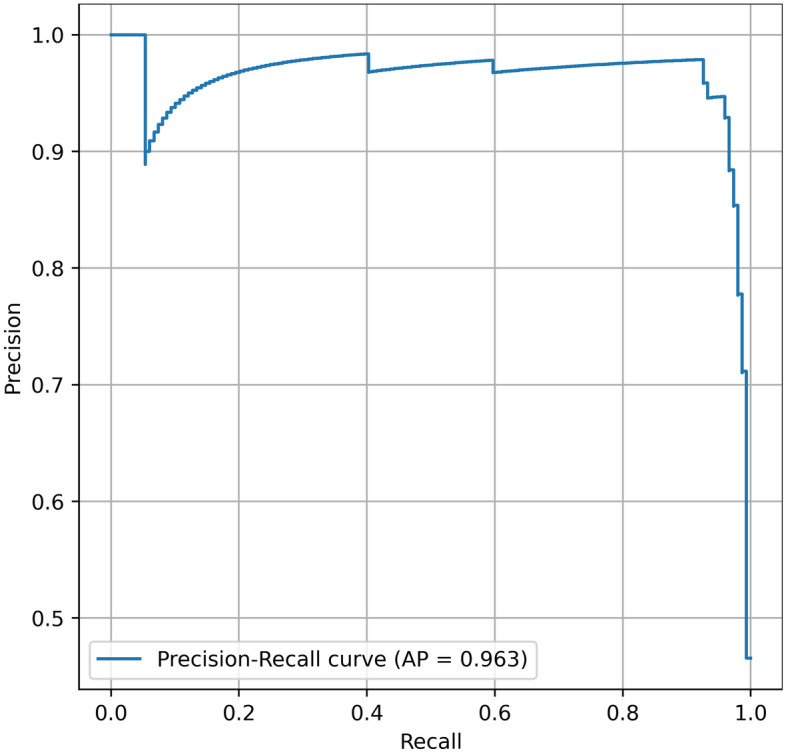
Precision-Recall-curve.

**Figure 4:**
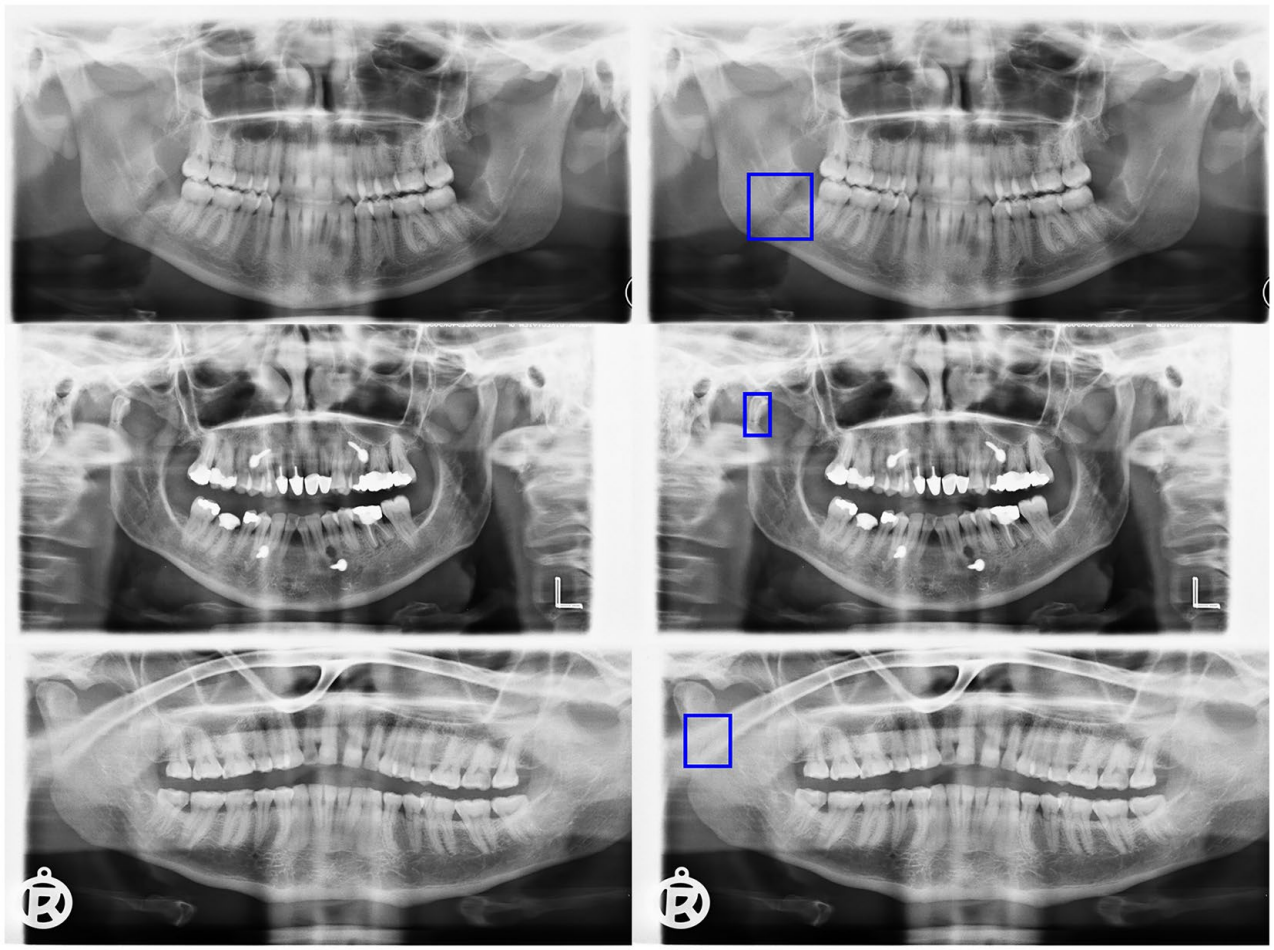
False positive predictions. The model predicted bounding boxes in areas without fractures.

**Figure 5:**
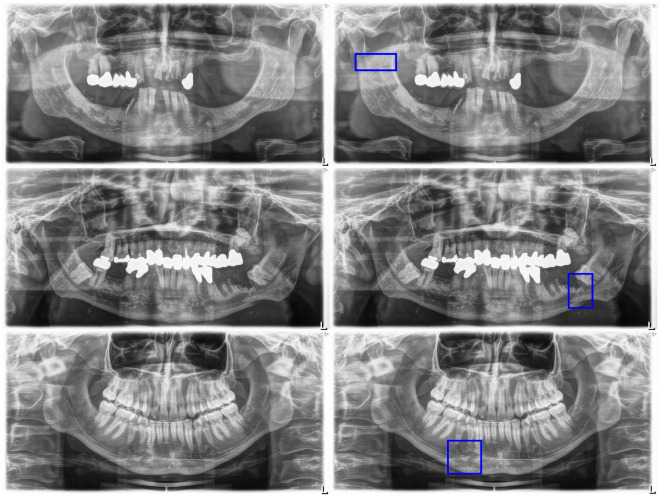
False negative predictions. The model failed to predict bounding boxes in areas with fractures.

Table 1 summarises the detection performance (fracture/non-fracture) of the Faster R-CNN with Swin-Transformer backbone on the test set. The model achieved a precision of 0.935, recall of 0.960 and F1-score of 0.947. The AUC and AP were 0.977 and 0.963, respectively (Figs. 2, 3). Examples of false positive predictions and false negative predictions are shown in Figs. 4 and 5.

**Table 1:** Binary detection metrics of mandibular fractures on panoramic radiographs.

Precision	Recall	F1-score	AUC	AP
0.935	0.960	0.947	0.977	0.963

*AUC* area under the curve receiver operating characteristics curve, *AP* precision-recall curve.

**Figure 6:**
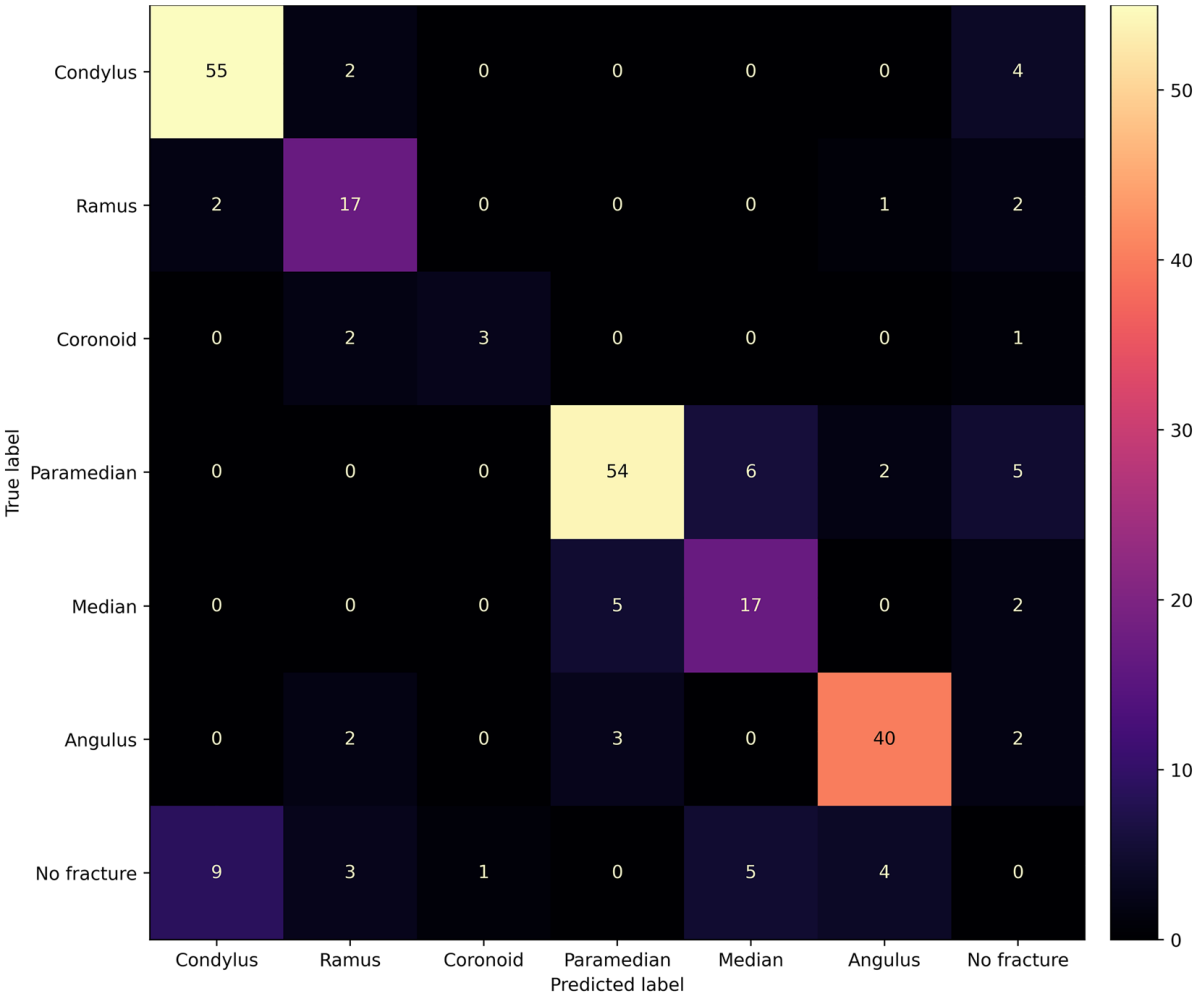
Confusion matrix illustrating the multi-class detection results of mandibular fractures (instance level) on PR. Instance level analysis takes multiple fractures into consideration on each OPG. True negatives were excluded so as not to confuse instance level analysis with image level analysis.

The confusion matrix in Fig. 6 illustrates the multi-class detection performance on fracture subtypes. The different subtypes were detected and classified with F1 scores ranging from 0.6 to 0.87 (Table 2). The lowest detection sensitivity was realized for coronoid fractures (three out of six), whereas the highest sensitivity was achieved for condyle fractures (55 out of 61).

**Table 2:** Multi-class detection metrics of mandibular fractures on panoramic radiographs.

	Precision	Recall	F1 score
Condyle	0.83	0.90	0.87
Ramus	0.65	0.77	0.71
Coronoid	0.75	0.50	0.60
Paramedian	0.87	0.81	0.84
Median	0.61	0.71	0.65
Angle	0.85	0.85	0.85

## Discussion

Mandibular fractures are one of the most frequent facial traumas in oral and maxillofacial surgery^1^. Surgeon-related factors such as fatigue or inadequate training increase the radiographic misinterpretation, leading to more extensive complications, including malocclusion, non-union, osteomyelitis, haemorrhage, and airway obstruction^10–12^. An automated assistance system for the clinician may serve as a second opinion. The Faster R-CNN and Swin-Transformer introduced in this study is a promising tool for detecting mandibular fractures on PR that are most often acquired as a first-level imaging technique in facial trauma patients.

Two studies have recently applied CNNs to detect mandibular fractures on PR^10,21^. Warin et al. applied DenseNet-121 and ResNet-50 to classify PR as PR with and PR without fractures. Both classification models achieved an F1 score and an AUC of 100%. In another experiment, Warin et al. used Faster R-CNN and YOLOv5 to detect mandibular fractures on PR. For the two investigated models, a precision of 87.94% and 86.12%, recall of 83.58% and 92.23%, and F1 score of 90.67% and 89.07% were reported^10^. In comparison, Son et al. used YOLOv4 with multi-scale luminance adaptation transform and single-scale luminance adaptation transform to detect mandibular fractures on PR. The reported F1 score ranged from 0.751 to 0.875^21^.

Warin et al. reported a higher recall and F1 score but lower precision compared to Son et al. However, a direct comparison of these previous studies should be regarded with caution. The performance of the CNNs and transformers is highly dependent on the dataset, the hyperparameters, and the architecture itself^22^. Warin et al. used 1710 PR, whereas Son et al. used only 420 PR. Warin et al. trained the Faster R-CNN for 20.000 iterations, with a 0.025 learning rate, 1882 epochs and a batch size of 128 images. A learning rate of 0.01, 200 epochs and a batch size of eight were used for YOLOv5. In contrast, Son et al. trained the YOLOv4 for 12.000 iterations with a batch size of 64 and a learning rate of 0.0001. Furthermore, the data representativeness was unclear. For these reasons, a direct comparison is misleading^10,21^.

In the current study, the Faster R-CNN with Swin-Transformer achieved an F1 score of 0.947 and an AUC of 0.977. Considering binary detection, the model had six false negative predictions and ten false positive predictions. The optical inspection of the false positive predictions showed that artefacts and superimpositions were misinterpreted as fractures. Furthermore, the different fracture subtypes were detected with varying accuracies. Fractures with the highest incidence, i.e. angle fractures, paramedian fractures and condylar fractures, were detected with an F1 score of 0.85, 0.84 and 0.87, respectively. In comparison, coronoid fractures had a significantly lower F1 score of 0.60. All in all, the detection performance was directly correlated with the available instances for training. The increase of annotated coronoid fractures may lead to better performance.

Although the results were promising, there are some limitations to this study. The reported study is limited by its monocentric design resulting in a database consisting of the local population. The PRs were acquired with only two different devices and did not regard clinical settings in which PR may be acquired with different scanners. Furthermore, the Faster R-CNN with Swin-Transformer was strictly confined to the employed train- and test set and may perform worse in real-world scenarios. In future studies, comparative studies are required to answer the choice of models more objectively. Multi-centred extension of labelled data is needed to increase the model’s robustness and generalizability^22^. Prospective studies are desired to evaluate the diagnostic accuracy in a clinical setting.

In conclusion, the Faster R-CNN and Swin-Transformer form a promising foundation for further developing automatic detection of fractures on PRs. Deep learning-based assistance of clinicians may reduce the misdiagnosis and hence severe complications.

## Data Availability

The data used in this study can be made available from the corresponding author within the regulation boundaries for data protection.
